# Supplementary figures and images for: Perturbation of BRMS1 interactome reveals pathways that impact metastasis
Source: PLoS One. 2021 Nov 17;16(11):e0259128. doi: 10.1371/journal.pone.0259128 (PMC8598058; doi:10.1371/journal.pone.0259128)

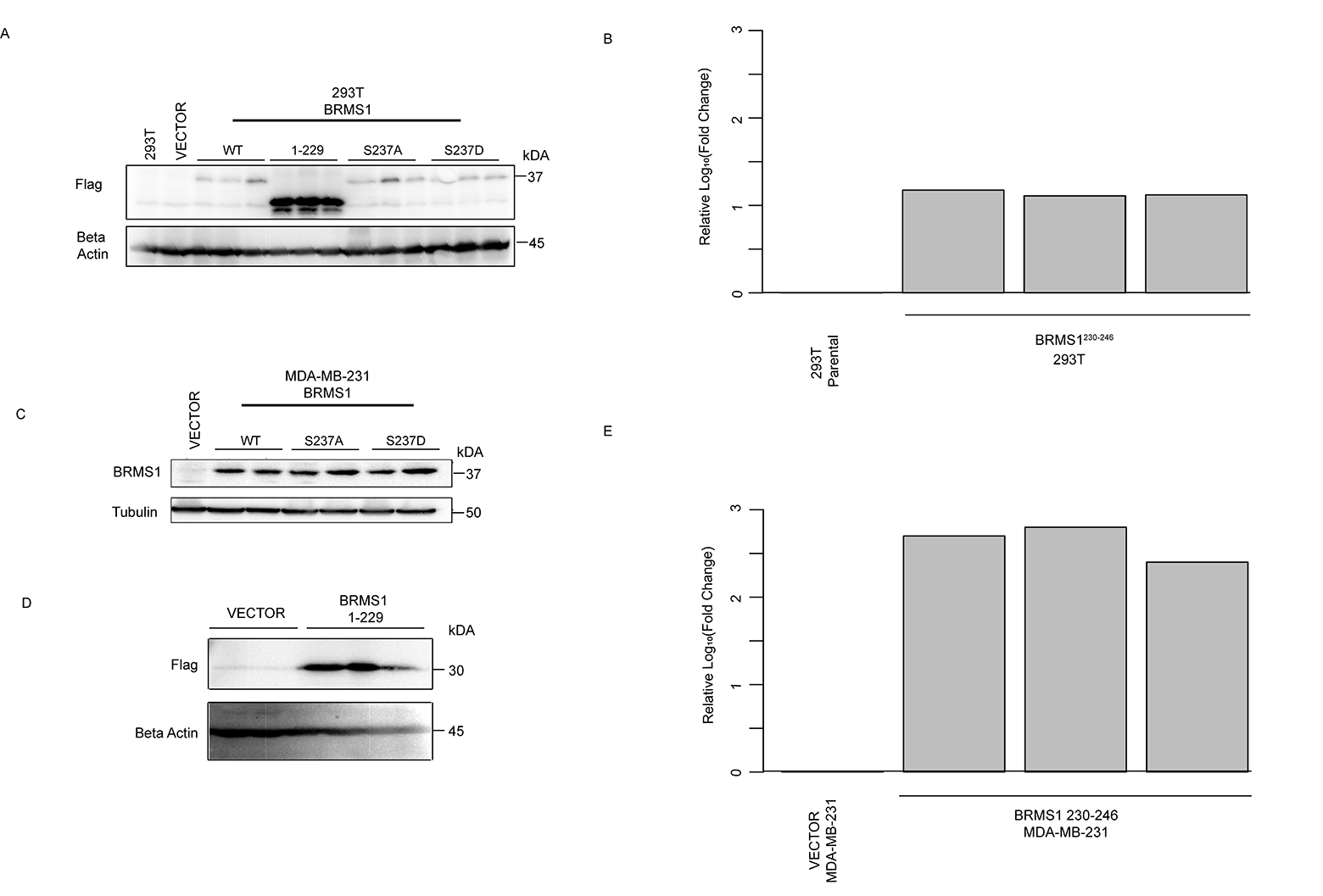

Supplement: S1 Fig — Expression of BRMS1WT, BRMS11-229, and phospho-mutants compared to parental and vector control 293T cells. (B) qPCR quantification of BRMS1230-246 expression in 293T cells. (C) Expression of BRMS1WT and phospho-mutants compared to vector control MDA-MB-231 cells. (D). Expression of BRMS11-229 within MDA-MB-231 cells. (E) qPCR quantification of BRMS1230-246 expression in MDA-MB-231 cells. (TIF) [file pone.0259128.s001.tif]

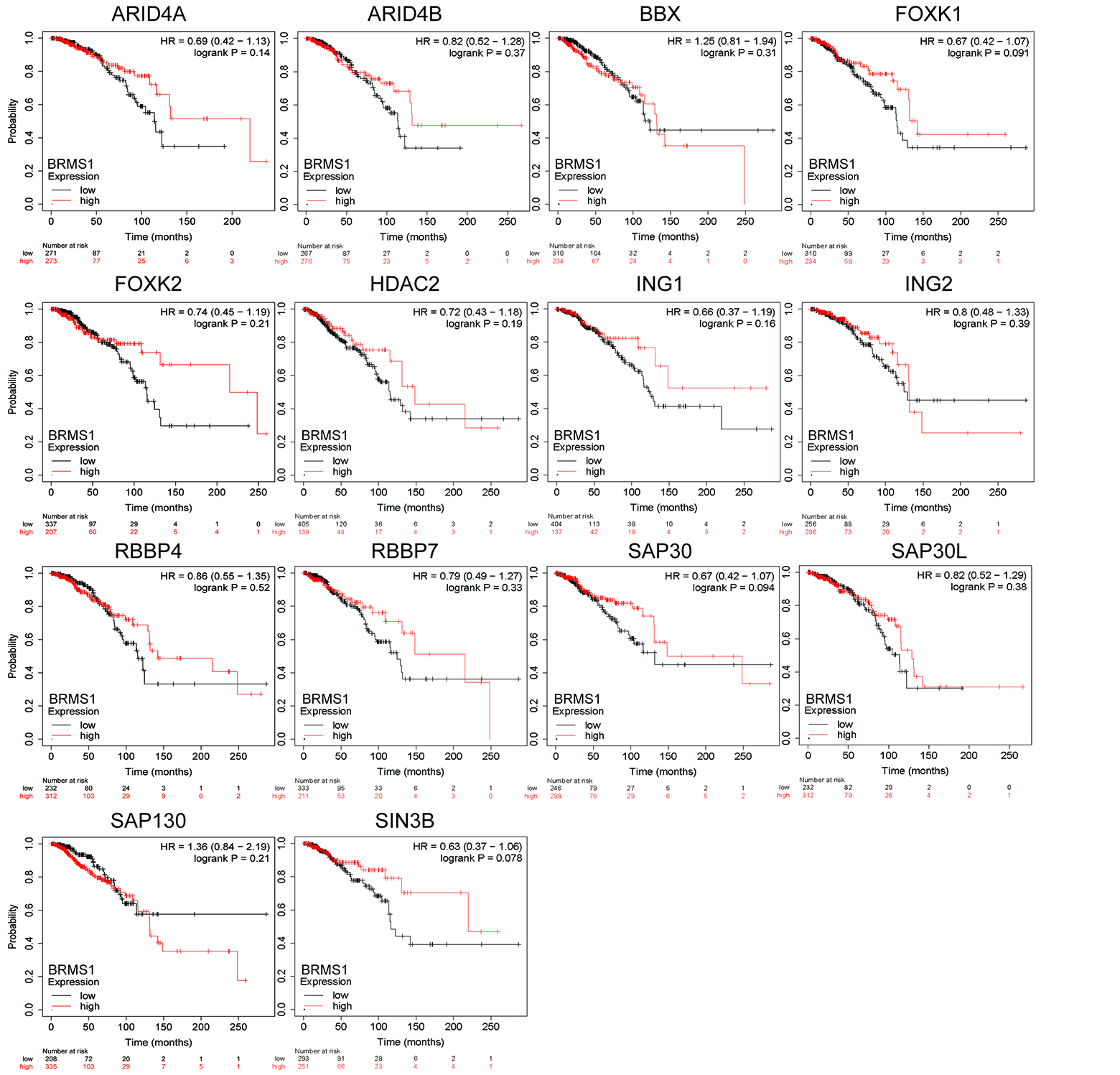

Supplement: S2 Fig — BRCA data was mined in which SIN3/HDAC members were separated by median expression into those greater than the median BRMS1 expression was then examined within these patients for overall survival, with high BRMS1 (indicated by Red) and low BRMS1 (indicated by black) were accounted for. This was completed in KM Plotter. (TIF) [file pone.0259128.s002.tif]

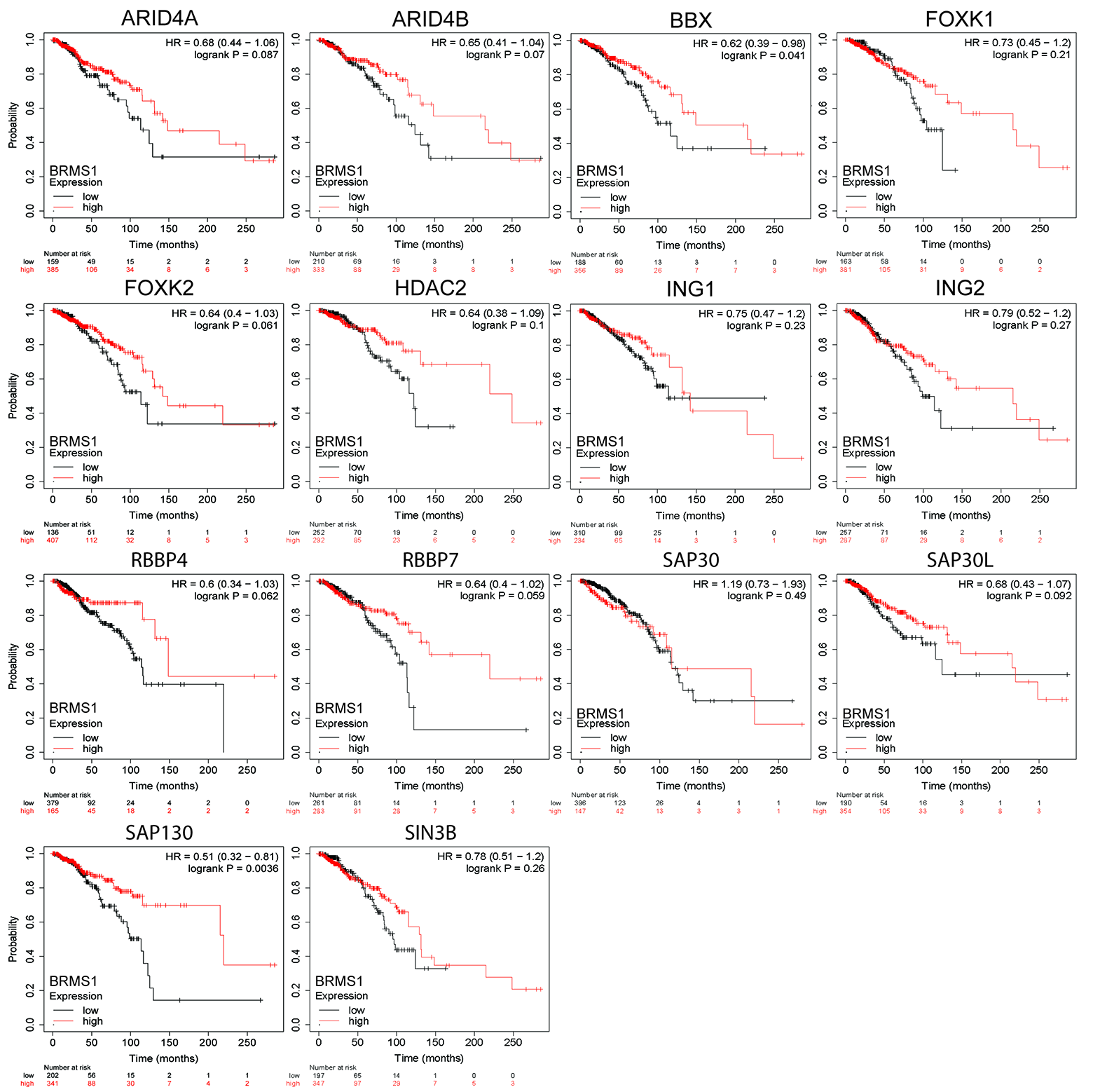

Supplement: S3 Fig — BRCA data was mined in which SIN3/HDAC members were separated by median expression into those less than the median BRMS1 expression was then examined within these patients for overall survival, with high BRMS1 (indicated by Red) and low BRMS1 (indicated by black) were accounted for. This was completed in KM Plotter. (TIF) [file pone.0259128.s003.tif]
