# Supplementary material for: Perturbation of BRMS1 interactome reveals pathways that impact metastasis
Source: PLoS One. 2021 Nov 17;16(11):e0259128. doi: 10.1371/journal.pone.0259128 (PMC8598058; doi:10.1371/journal.pone.0259128)
Supplement: S3 Table — (DOCX) [file pone.0259128.s006.docx]

**S3 Table. SIN3A known interactors and validation method.**

| **Protein** | **Validation by Western Blot** | **Validation by Mass Spectrometry** |
| --- | --- | --- |
| ARID4A | [42,43] | [13,44] |
| ARID4B | [45] | [13,45,46] |
| SAP130 | [45] | [13,44,45] |
| HDAC2 | [47-49] | [13,44,50] |
| RBBP4 | [51,52] | [13,46,53] |
| RBBP7 | [54] | [13,44,46,55] |
| SUDS3 | [45] | [13,44,55] |
| BRMS1L | [56] | [13,44,46] |
| SAP30 | [57,58] | [13,44,46] |
| BRMS1 | [11,22] | [13,44] |

Previously validated SIN3A interactors either by Western Blot or Mass Spectrometry.
