# Supplementary material for: Perturbation of BRMS1 interactome reveals pathways that impact metastasis
Source: PLoS One. 2021 Nov 17;16(11):e0259128. doi: 10.1371/journal.pone.0259128 (PMC8598058; doi:10.1371/journal.pone.0259128)
Supplement: S1 Data — (DOCX) [file pone.0259128.s011.docx]

**Primers used in this study**

1. **Primers used to construct vectors expressing Halo tagged BRMS1 and BRMS1 mutants**

For cloning Halo-BRMS1 pcDNA5/FRT:

BRMS1 SgfI F 5’- CAGGCGATCGCCATG CCT GTC CAG CCT CCA AG- 3’

BRMS1 PmeI R (with stop) 5’- CAGGTTTAAACTCA AGG TCC ATC CGA TTT TCT CTT CTG AG - 3’

For cloning Halo-BRMS1 S237A pcDNA5/FRT:

BRMS1 SgfI F  as above

BRMS1 S237A PmeI R 5’- CAGGTTTAAACTCA TCA AGG TCC ATC CGA TTT TCT CTT CTG AGG GGC CAC AGC TGC CCT AGC CTT TTT GAT G - 3’

For cloning Halo-BRMS1 S237D pcDNA5/FRT:

BRMS1 SgfI F as above

BRMS1 S237D PmeI R 5’- CAGGTTTAAACTCA TCA AGG TCC ATC CGA TTT TCT CTT CTG AGG GTC CAC AGC TGC CCT AGC CTT TTT GAT G - 3’

For cloning Halo-BRMS1 1-229 pcDNA5/FRT:

BRMS1 SgfI F as above

BRMS1 229 PmeI R 5’- CAGGTTTAAACTCA GAT GGC TGT CCA GTC CTC CAG - 3’

1. **Synthetic duplex DNA sequence used to clone BRMS1 230-246 into pcDNA5/FRT**

5’ – CAG GCG ATC GCC AAA AAG GCT AGG GCA GCT GTG TCC CCT CAG AAG AGA AAA TCG GAT GGA CCT TGA GTTTAAACGAC – 3’
